# Fit for Service: Preparing Residents for Neurointensive Care with Entrustable Professional Activities: A Delphi Study

**DOI:** 10.1007/s12028-023-01799-x

**Published:** 2023-07-27

**Authors:** Moritz L. Schmidbauer, Severin Pinilla, Stefan Kunst, Anne-Sophie Biesalski, Julian Bösel, Wolf-Dirk Niesen, Patrick Schramm, Katja Wartenberg, Konstantinos Dimitriadis, the IGNITE study group

**Affiliations:** 1 Department of Neurology, LMU University Hospital, LMU Munich, Munich, Germany; 2 University Hospital for Old Age Psychiatry and Psychotherapy, University of Bern, Bern, Switzerland; 3 Institute for Medical Education (IML), University of Bern, Bern, Switzerland; 4 Department of Neurology, Ruhr-Universität Bochum, St. Josef Hospital, Bochum, Germany; 5 Department of Neurology, Heidelberg University Hospital, Heidelberg, Germany; 6 Department of Neurology and Neurophysiology, University Medical Center Freiburg, Freiburg, Germany; 7 Department of Neurology, Universitätsklinikum Giessen und Marburg, Standort Giessen, Justus-Liebig-University, Giessen, Germany; 8 Department of Neurology, University of Leipzig, Leipzig, Germany; 9 Institute for Stroke and Dementia Research (ISD), LMU University Hospital, LMU Munich, Munich, Germany

**Keywords:** Intensive care unit, Postgraduate medical education, Neurointensive care, Entrustable professional activities, Delphi study

## Abstract

**Background:**

Although the relevance of neurointensive medicine and high-quality training of corresponding physicians is increasingly recognized, there is high heterogeneity in the nature, duration, and quality of neurointensive care curricula around the world. Thus, we aimed to identify, define, and establish validity evidence for entrustable professional activities (EPAs) for postgraduate training in neurointensive care to determine trainees’ readiness for being on-call.

**Methods:**

After defining EPAs through an iterative process by an expert group, we used a modified Delphi approach with a single-center development process followed by a national consensus and a single-center validation step. EPAs were evaluated by using the EQual rubric (Queen’s EPA Quality Rubric). Interrater reliability was measured with Krippendorff’s *α*.

**Results:**

The expert group defined seven preliminary EPAs for neurointensive care. In two consecutive Delphi rounds, EPAs were adapted, and consensus was reached for level of entrustment and time of expiration. Ultimately, EPAs reached a high EQual score of 4.5 of 5 and above. Interrater reliability for the EQual scoring was 0.8.

**Conclusions:**

Using a multistep Delphi process, we defined and established validity evidence for seven EPAs for neurointensive medicine with a high degree of consensus to objectively describe readiness for on-call duty in neurointensive care. This operationalization of pivotal clinical tasks may help to better train clinical residents in neurointensive care across sites and health care systems and has the potential to serve as a blueprint for training in general intensive care medicine. It also represents a starting point for further research and development of medical curricula.

**Supplementary Information:**

The online version contains supplementary material available at 10.1007/s12028-023-01799-x.

## Introduction

Design and implementation of postgraduate training curricula can be a major challenge, especially when dealing with emergencies and high levels of specialization and when performed as training on the job. This applies to intensive care and emergency medicine and even more so for neurointensive care because of the distinct time-critical nature of neurological emergencies. Trainees in these settings often feel overwhelmed with the workload, the working environment, and the emotionally challenging life and death decisions [1].

In recent decades, medical education has evolved from content-based teaching catalogs and time-based curricula to competency-based medical education. Regarding critical care, the Competency-Based Training Programme in Intensive Care Medicine in Europe (CoBaTrICE) was established as an international standard in 2006 [2]. Moreover, the European Society of Intensive Care Medicine has introduced a European Diploma in Intensive Care Medicine in order to standardize education [3]. However, a US-based survey among neurocritical care fellowship program directors revealed significant heterogeneities in neurocritical care training as well as in the process of verifying the attainment of a competency level [4]. To tackle this issue, the Accreditation Council for Graduate Medical Education (ACGME) defined milestones as operationalized learning goals for neurocritical care in 2022 [5]. Some of the defined milestones (e.g., “Consistently demonstrates technical skill to successfully and safely perform and interpret invasive procedures”), despite involving clinical tasks, do not represent discrete, observable, and specific work processes on a neurointensive care unit (NICU), whereas others (e.g., “Demonstrates leadership and mentorship in applying ethical principles”) describe competences and abilities of persons rather than observable clinical tasks [5]. Although competency-based curricula focus more on outcome, some clinical educators have raised concern, especially because abstract competencies often appear too detached from clinical work, and patient safety ultimately depends on matching expected and entrusted clinical competence [6–9]. To further close the gap between abstract learning goals and concrete clinical tasks a trainee must be entrusted with, Ten Cate and colleagues introduced the concept of entrustable professional activities (EPAs) [10, 11]. An EPA is defined as “a unit of professional practice that can be fully entrusted to a trainee, once he or she has demonstrated the necessary competence to execute this activity unsupervised” [9]. Although “unit” could be any concrete task that contributes to patients’ health, EPAs sometimes include a bundle of tasks for reasons of practicality. According to Ten Cate, “the purpose of using EPAs is to operationalize competency-based medical education through a stepwise and safe engagement of trainees in clinical practice—linking progressive proficiency to progressive autonomy in patient care” [9]. Thus, EPAs are units of work that focus on observable outcomes of care, in contrast to milestones, which tend to focus on trainee abilities [12].

Although EPAs have become increasingly popular in several health care professions, with publication numbers rising higher each year, there are only a few defined and validated EPAs for critical care, and to our knowledge, there are no EPAs for neurointensive care medicine. Most published EPAs in the critical care setting refer to pediatric intensive care unit (ICU) curricula or general anesthesiologic curricula [13–16]. Often, EPAs found in the literature are organized by management of a medical condition or disease rather than representing an observable work process, raising questions regarding their usefulness in clinical supervision.

The aim of our study was to identify, define, and establish validity evidence for potential EPAs for postgraduate training in neurointensive care. We focus on residents’ readiness for working on a neurointensive care ward and for being on-call on weekends or during night shifts after onboarding. We used a modified Delphi process including a national consensus among key stakeholders for training clinical residents in neurointensive care.

## Methods

### Setting

In Germany, postgraduate neurologic training is regulated by the federal medical association. It includes six mandatory months of (neuro-)ICU training for every neurology resident. To formally subspecialize in neurointensive care medicine, 18 additional months of ICU training after specialization in neurology/neurosurgery are required (which is comparable to fellowship programs in the United States) [17]. Attending physicians or senior residents are typically responsible for clinical supervision of trainees. Workplace-based postgraduate training curricula in Germany are predominantly designed as “training on the job,” with little or no other instructional formats [18]. The default level of expected competence for neurologists during residency training and all ICU subspeciality trainees is to manage neurological intensive care patients independently and do ICU shifts on weekends or at night.

### Study Design and Study Participants

To identify, define, and establish validity evidence for EPAs for postgraduate training in neurointensive care, we performed a modified Delphi study in a three-step approach: (1) a single-center development process (non-Delphi process), (2) a national multicenter consensus process (Delphi study), and (3) a single-center validation process. The first and third steps were performed at the Department of Neurology at Ludwig Maximilians University (LMU). For the second step, leading German neurointensivists and members of the German Society for Neurointensive Care and Emergency Medicine (German: *Deutsche Gesellschaft für Neurointensiv- und Notfallmedizin* [DGNI]) and of the Initiative for German Neurointensive Trial Engagement (IGNITE) network were involved.

The expert group consisted of five clinicians with different neurointensive care experiences, profound educational expertise, and a clinical training background. Four of five were neurologists (three attendings, one fifth-year resident), one was a psychiatrist (attending in psychiatry with 2 years of NICU experience). Four of them had additional specific medical education training (Master of Education, doctoral or postdoctoral qualification in medical education). All five were familiar with the EPA concept, and two had previous experience in developing EPAs.

To be as close as possible to clinical and educational everyday practice, we included early trainees in the first Delphi round who had just completed their NICU rotation or who were currently in the process of doing so. For the next round, and after reaching a consensus on critical points and incorporating the corresponding results of the first round, we included experienced neurointensive care physicians and educators who, as part of their work for the DGNI, were already involved in the design of the national competence catalog for neurointensive medicine and the development of NICU training curricula. All six participants in this round had more than 10 years’ working and teaching experience, and five of them also had more than 10 years’ NICU working experience. The last round of validation was conducted by two experienced neurointensive care physicians with no previous involvement in the EPA development process who were introduced to the EPA concept but had no personal experience with EPAs.

### Study Procedure

**Fig. 1:**
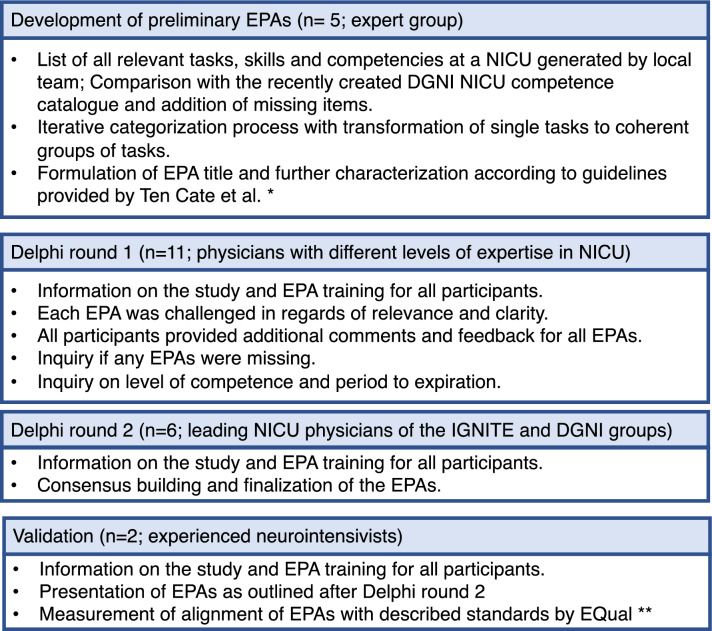
Delphi process and EPA development. DGNI *Deutsche Gesellschaft für Neurointensiv- und Notfallmedizin*, EPAs entrustable professional activities, EQual Queen’s EPA Quality Rubric, IGNITE Initiative for German Neurointensive Trial Engagement, NICU neurological intensive care unit. *Ref. [9]. **Ref. [19]

The detailed process of EPA development is shown in Fig. 1. In the first step, the group outlined all tasks, skills, and competences necessary in a NICU. In the second step, the list was compared to the recently validated competence catalog of the DGNI (which was validated in a detailed perennial process with the participation of different committees and professional societies) [17]. Missing elements were supplemented, and duplicates were resolved. The final list was used to identify and generate candidate EPAs iteratively. Once the titles and content of the EPAs had been outlined, they were drafted in detail based on the recommendation by Ten Cate et al. [9]. During the first Delphi round, participants were familiarized with the EPA concept. Then all candidate EPAs were presented to all participants, and feedback was requested concerning relevance, clarity, and content within a 4-week period. All participants had the chance to add or suggest missing elements. Finally, the level of competence and the period of expiration were discussed until the group reached consensus. After incorporating suggestions made by the group, the adapted EPAs were piloted by the leading NICU physicians on a national level in a second Delphi round. The last step was performed to establish validity evidence for the final detailed EPAs list with their descriptions based on the EQual rubric (Queen’s EPA Quality Rubric) [19].

### Statistical Analysis

For the validation round, and based on the scoring of the EQual questionnaire, an interrater reliability for the two raters was calculated. The questionnaire consists of 14 Likert items, each with five answer options (scales were item specific and were used as originally published [19]). In order to account for nonparametric distribution of ratings, we used Krippendorff’s *α* to calculate interrater reliability [20]. A coefficient for the whole data set (all items for all seven EPAs) and individual coefficients for each EPA were calculated. For each item of the scale and each EPA, the scores of both raters were averaged. Then average EPA scores (based on all 14 items) of the scale were calculated.

### Ethical Approval

Because all participants declared their consent to participate by voluntarily taking part in the study and all data were collected anonymously, the study was exempt from additional formal ethical review in consultation with the local ethics committee of LMU Munich.

## Results

Information on the number of participants and demographics, including educational and clinical experience, in the three rounds of the process is presented in Table 1.

**Table 1:** Demographics

	Expert group	Delphi round 1	Delphi round 2	Delphi round 3
*n*	5	11	6	2
Sex (female/male)	1/4	6/5	2/4	1/1
Age in years (range)	30–66	27–32	35–52	36–40
Medical education background^a^ (*n*)	4	1	5	1
Teaching experience in years (range)	5–36	1–5	10–21	10–11
Work experience in years (range)	8–36	1–8	10–24	10–11
NICU experience in years (range)	2–30	0.1–2	2.5–18	3–4

*NICU* neurological intensive care unit

^a^Specific medical education training (e.g., Master of Medical Education, Master of Education, doctoral or postdoctoral qualification)

### Development of EPAs

The detailed process of EPA development in the expert group is illustrated in Supplement 1. After consolidating the two lists (first list outlined by the expert group and second list derived from the DGNI-NICU competence catalog), 108 learning goals remained. The subsequent categorization process resulted in seven EPA titles (Table 2).

**Table 2:** EPA titles

EPA titles
1. Identifying and conducting appropriate clinical (clinical–neurological) examination methods to assess NICU patients
2. Performing specialized neurological diagnostic or therapeutic procedures on NICU patients
3. Performing general ICU-specific diagnostic and therapeutic procedures
4. Recognizing an emergency situation, initiating stabilization of patients, and reaching out for help
5. Transporting a NICU patient outside the NICU
6. Initial general management of NICU patients
7. Handing over NICU patients

*EPA* Entrustable professional activity, *ICU* intensive care unit, *NICU* neurological intensive care unit

After agreement was reached on the content of EPA titles and which of the 108 items were subordinate to which of the EPAs, more precise definitions and limitations were worked out in the next step. An example of a detailed EPA is illustrated in Table 3. All seven EPAs can be found in Supplement 2.

**Table 3:** EPA 5 in detail

	EPA 5
Title	Transporting a NICU patient outside of the NICU
Description (specification and limitations)	Preparing an intrahospital transport of an ICU patient (premedication if indicated, appropriate monitoring, preparation of useful medication or equipment for transport)
	Conducting the transport (including handling of equipment, e.g., manual ventilation if indicated)
	Dealing with emergencies during transport
	Handover of the patient to other medical professionals
	Communicating with the team and obtaining help are part of this EPA
Required knowledge, skills, attitudes, and experiences (knowledge, skills, attitude)	Knowledge of suitable algorithms for emergency situations
	Knowledge of the pharmacological properties of vasoactive substances, sedatives, and other emergency drugs frequently used
	Mastering use of equipment during transport
	Leading the team in an emergency situation
	Communication with patients and the team
	Ability to hand over the most important medical information in a structured manner
Potential risks in case of failure	Risk of missing emergency equipment necessary to manage complications during transport
	Risk of harming the patient due to failure to use appropriate therapeutic measures or miscommunication (e.g., during handover)
	Risk of causing harm to patients
Most relevant competency domains (CanMEDS)	Medical expert
	Communicator
	Team worker
	Leader
	Professional
Information sources to assess progress and support summative entrustment	Direct observation of procedural skills
	Case-based discussions
	Entrustment-based discussions
Entrustment/supervision level expected	Levels 4 and 5
Time period to expiration if not practiced	2 years

*CanMEDS* Canadian Medical Education Directives for Specialists, *EPAs* entrustable professional activities, *ICU* intensive care unit, *NICU* neurological intensive care unit

### Delphi Rounds 1 and 2

All participants (100%, 11 of 11) of the first round agreed that the titles of the seven defined EPAs covered all aspects necessary for preparing residents for working in a NICU. Remarks and corrections mostly concerned specifications of content and limitations and, above all, differentiation from other EPAs. Only 2 of 11 proposed changes were related to entrustment level or time to expiration. An additional three participants commented that they felt uncertain regarding the entrustment level or expiration time. Similar to the previous round, all NICU experts of the second round confirmed the completeness of the EPAs in covering all elements important for preparing trainees. However, one participant recommended formulating a separate EPA to highlight its importance (communication, team, and error management). In further discussions, the group decided against this proposal on the grounds that this is more of a competency than a task and thus is part of all EPAs. Further discussions focused on the granularity of the descriptions, especially because ICUs differ in structure, equipment, and patient population. Given every EPA can further be specified according to the characteristics of the local NICU, agreement could be reached for the form presented. Finally, consent among all experts was also reached on entrustment level and time period to expiration.

### Validation

Using the EQual rubric, two experienced neurointensivists collectively rated all EPAs with a mean (SD) overall score of 4.81 (± 0.16). EPA 3 reached the highest mean score with 5.0, and EPA 6 reached the lowest mean score with 4.5 (± 0.62). Mean scores for all seven EPAs are presented in Table 4. The minimum rating for an item in our study was 3. Only 7 of 98 items scored 3 (regarding single rater scores) on the Likert scale (EPA 1: items 2 and 4; EPA 4: item 2; EPA 5: item 5; EPA 6: items 2, 3, and 4). A more detailed presentation of scores per item and EPA can be found in Supplement 3. Krippendorff’s *α* score for interrater reliability was 0.80 for the whole data set. The individual coefficient scores for all EPAs are presented in Table 4. The lowest agreement was measured for EPA 1 with 0.51, whereas EPAs 2, 3, and 7 had perfect interrater reliability. As proposed by the authors and developers of the EQual rubric, we recalculated the interrater reliability by excluding items 2, 6, and 14 of the original scale leading to higher values for Krippendorff’s *α* for EPA 1 (0.70) and EPA 5 (0.73) (Supplement 4) [19].

**Table 4:** Interrater reliability and EQual score

EPA titles	Interrater reliability (Krippendorff’s *α*)	Validity (EQual score)
1. Identifying and conducting appropriate clinical (clinical–neurological) examination methods to assess NICU patients	0.51	4.64 (± 0.50)
2. Performing specialized neurological diagnostic or therapeutic procedures on NICU patients	1.0	4.93 (± 0.27)
3. Performing general ICU-specific diagnostic and therapeutic procedures	1.0	5.0 (± 0.0)
4. Recognizing an emergency situation, initiating stabilization of patients, and reaching out for help	0.99	4.89 (± 0.40)
5. Transporting a NICU patient outside the NICU	0.59	4.86 (± 0.36)
6. Initial general management of NICU patients	0.73	4.5 (± 0.62)
7. Handing over NICU patients	1.0	4.86 (± 0.36)
Total	0.80	4.81 (± 0.16)

Using the EQual rubric, two experienced neurointensivists collectively rated all EPAs. Krippendorff’s *α* score for interrater reliability and means (± standard deviations) for EQual scores are presented

*EPAs* entrustable professional activities, *EQual* Queen’s EPA Quality Rubric, *ICU* intensive care unit, *NICU* neurological intensive care unit

## Discussion

In this three-step study, we used an iterative process combined with a modified Delphi protocol to generate seven EPAs with high validity evidence and interrater reliability for the training of residents in neurointensive care. Moreover, all NICU experts valued flexibility of the general EPA concept and its potential to be adapted to local circumstances.

Although the relevance of neurointensive medicine and high-quality training of corresponding physicians is increasingly recognized, there is high heterogeneity in the nature, duration, and quality of neurointensive care curricula around the world [4, 18, 21–23]. In a recent German survey among residents and program directors, a significant number of residents reported not having a written introductory concept for the ICU [18]. Although there are operationalized learning goals, such as the ACGME’s milestones of neurocritical care [5], there are issues with the applicability of these competency-based frameworks in clinical practice, such as the ongoing debate on the quantity of required procedures to gain proficiency in practical skills [4, 18, 23]. However, an arbitrary number of repetitions does not help program directors to decide whether they can entrust a resident with tasks necessary for a night or weekend shift without direct supervisions. More importantly, it does not provide any opportunity for formative feedback to help residents become competent. Our study, and the here defined EPAs, could enhance the discussion among neurointensivists globally in redefining their curricula and move toward outcome-based educational concepts. Development, agreement, and validation of EPAs, however, is just the first step. It has to be followed by development of adequate assessment methods, design of EPA-based curricula, and evaluation of these curricula in multidimensional mixed-methods studies.

The overall high level of agreement within the Delphi rounds objectified by the EQual rubric indicated that the here presented EPAs appear to cover the most relevant entrustable working packages in a NICU. Yet Krippendorff’s *α* for EPAs 1 and 5 was low, indicating reduced interrater reliability. This was mostly related to discrepancies in item 2, 6, or 14 of the EQual rubric. In the original validation study for the EQual rubric, these items performed badly regarding their psychometric properties and interrater variability as well [19]. A post hoc elimination of those three items in the aforementioned study reduced the variance significantly. In the end, authors therefore proposed to eliminate those for the instrument [19]. Similarly, once removed in our study, Krippendorff’s *α* for EPAs 1 and 5 increased and was comparable with that for the remaining EPAs (Supplement 4).

The discussions, especially among the experts in the second round, highlighted the tightrope walk between a small number of EPAs with value for clinical practice on the one hand and sufficient granulation of the content on the other hand. In that regard, EPAs 2, 3, 4, and 6 were debated the most. In this study, an agreement on that matter could be reached among both residents in training and experienced neurointensivists. The somewhat broader definition could complicate monitoring or assessment of the corresponding elements of the EPA and should be examined more closely in clinical practice. If it turns out during implementation that a more detailed description is necessary to evaluate entrustment, one could define sub-EPAs or observable practice activities within the here defined EPAs according to the work of Emke et al. [24]. However, the presented concept offers the possibility of defining the relevant subcategories for each clinical center and ICU individually according to local circumstances. Moreover, it makes the tasks for trainees during a shift more transparent and could therefore make entrustment more factual. Finally, across different educational systems, learning objectives are acquired in different phases of education (learning objectives can be part of either residency or fellowship programs). Because EPAs are defined as units of work rather than abilities or levels of competence, they can be used for the corresponding level of supervision of each training phase with small adjustments. The EPAs we developed are primarily intended for residents. However, residents might not be expected to supervise trainees for all identified EPAs at the end of their training (i.e., level 5) in contrast to fellows. Thus, the level of supervision can be used to differentiate between training phases (residency vs. fellowship). Furthermore, the broadness (or granularity) of EPAs could differ (intubating a patient vs. managing a NICU) according to training phase.

Given the overlap of the milestones formulated by ACGME and the EPAs proposed here, it is reasonable to assume that this framework is extensive and internationally applicable [5, 25]. Although milestones reflect trainees’ abilities, whereas EPAs describe specific tasks, some of the milestones defined by ACGME are very similar to the EPAs proposed here. Moreover, most of the content of ACGME’s milestones can be found in the description of the “knowledge, skills, attitudes, and experiences” section of the EPAs described here. For example, the ACGME milestone “Demonstrates skill in performing, managing, and interpreting invasive procedures. (Procedural, General Critical Care)—Patient Care 4a” is almost identical to EPA 3 (“Performing general ICU-specific diagnostic and therapeutic procedures”). Others, such as ACGME’s “Interpersonal and Communication Skills 4: Interprofessional and Team Communication,” are part of the described attitudes in some of the EPAs, such as in EPA 5 (see Table 3, “knowledge, skills, attitudes, and experiences” section). In a study comparing milestones with an EPA framework among surgical residents in the United States, high correlations were observed. However, the authors in this study concluded, that “EPAs may provide more timely and specific feedback than existing tools” [26]. Furthermore, it is potentially easier for clinical educators to allocate limited educational resources using an EPA framework in comparison to highly granular milestones [27]. Given the potential advantages of EPAs, systems with already established competency-based frameworks, such as the ACGME milestones, could be linked with EPAs to simplify the assessment of milestone achievement accordingly [27].

Although this is the first study to identify potential EPAs for NICU residency, other disciplines have started to publish their EPA-based residency programs or experiences with implementing EPA-based residency training programs [28–30]. Next steps for implementing EPA-based curricula in residency or fellowship programs would be to adapt the here proposed EPAs to local contexts, link them to existing competency-based learning objectives or milestones, develop or adapt the required workplace-based assessment tools, and finally design the corresponding teaching activities as well as the timeline of assessment (low stakes and high stakes) based on educational needs, feasibility, and health care system necessities. Future studies should explore the specific implementation context of neurocritical care residency training based on EPAs.

A strength of our study is the involvement of a diverse, highly experienced expert group that has already worked on a competency-based catalog, as well as doctors from different training levels for the Delphi process. Thereby, different perspectives (educational, clinical, trainee, and trainer) were included in evaluating the defined EPAs. Moreover, all participants in the Delphi process were trained regarding EPA background and concept. In addition, we accounted for the newly completed national competence catalog developed. Finally, the use of EQual allowed for a formalized evaluation with a well-validated and extensive tool.

Our results could be limited by the design of the Delphi process and the relatively small number of participants. However, from a constructivist perspective, we felt it was more important to make sure to capture key stakeholder perspectives. In our case, these included experts in NICU work, trainees, medical education experts, and existing literature. Other than in more traditional Delphi studies, we let the expert group define the EPAs and used the different Delphi rounds for feedback, adjustments, and validation rather than let Delphi study participants shape the EPAs. As a result, ideas could be lost by setting the frame for the group. However, as mentioned previously, the expert group had specific medical education expertise and used all available resources. Moreover, EPA development is a time-consuming process that most clinical active neurointensivists would struggle to shovel free. Therefore, our chosen approach seemed pragmatic and feasible. Another limitation could be the development of EPAs in a single-center setting. However, we counteracted this by including multicenter experts in the second Delphi round. Although the framework was developed in the context of the German national health care system, we believe that given the high degree of overlap between competency catalogs in international literature on the one side and the here presented EPAs on the other side, adapting the framework internationally is feasible and potentially benefits health care professionals globally.

## Conclusions

Our study presents the first set of well-defined EPAs for neurointensive care. These EPAs can be deployed across sites and health care systems. Thus, this framework could enhance a process of rethinking postgraduate NICU training and provide a starting point for designing further implementation studies, constructing suitable assessment methods, and designing EPA-based curricula and prospective real-world, outcome-based studies. In the meantime, it makes core clinical tasks transparent and could therefore help to advance training of residents or fellows in neurointensive care.

### Supplementary Information

Below is the link to the electronic supplementary material.

Supplement 1: Iterative EPA development by the expert group (DOCX 309 kb)

Supplement 2: Detailed description of all 7 EPA (DOCX 42 kb)

Supplement 3: Table with detailed scoring for the EQual instrument per Item and EPA (DOCX 17 kb)

Supplement 4: Table with values for the adjusted interrater reliability after removing items 2, 6 and 14 (DOCX 14 kb)
