# Supplementary figures and images for: Fit for Service: Preparing Residents for Neurointensive Care with Entrustable Professional Activities: A Delphi Study
Source: Neurocrit Care. 2023 Jul 27;40(2):645–53. doi: 10.1007/s12028-023-01799-x (PMC10959831; doi:10.1007/s12028-023-01799-x)

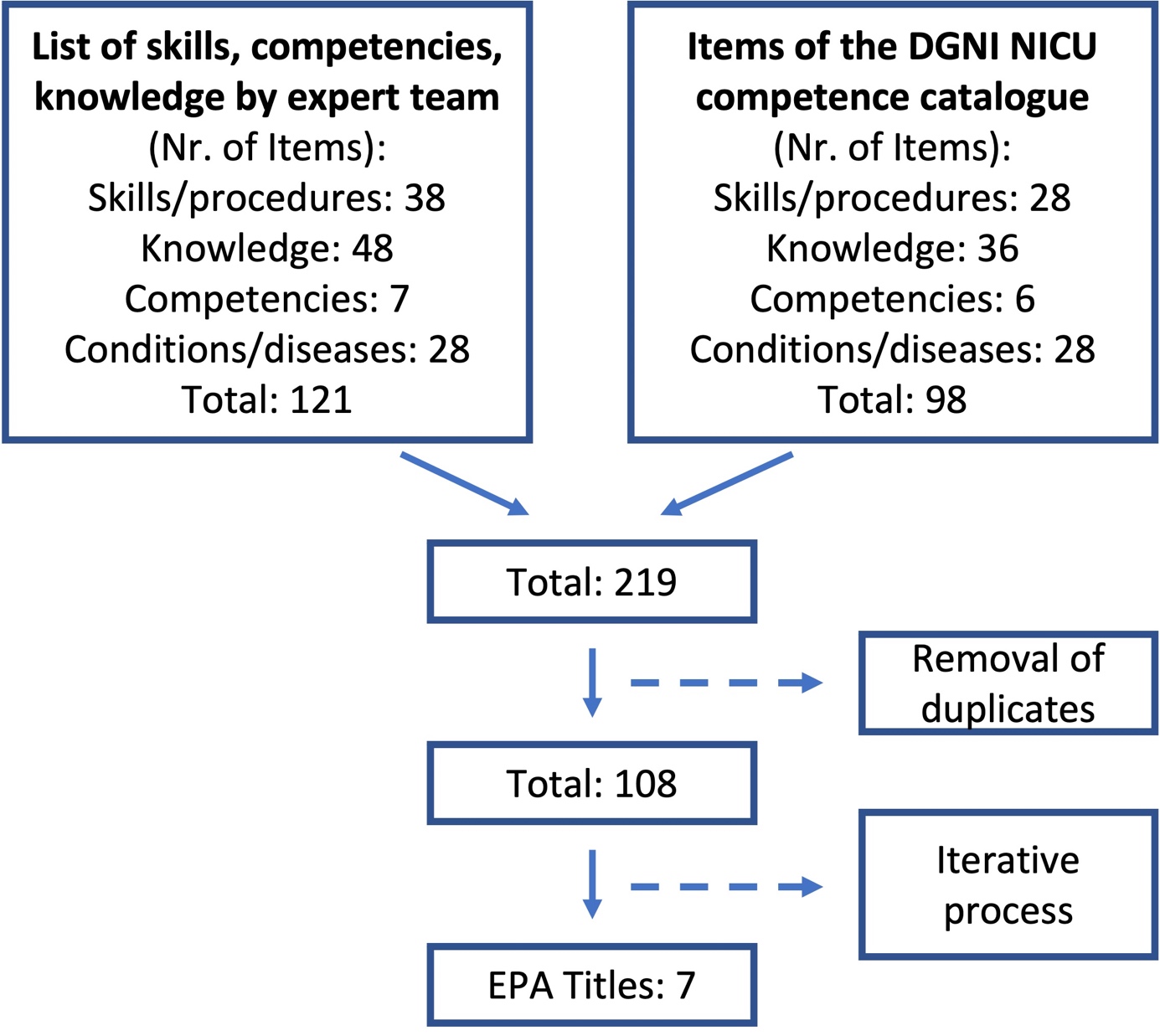


Iterative EPA development by the expert group

Supplement: Supplementary file 1 — Supplement 1: Iterative EPA development by the expert group (DOCX 309 kb) [file 12028_2023_1799_MOESM1_ESM.docx]
