# Supplementary material for: Fit for Service: Preparing Residents for Neurointensive Care with Entrustable Professional Activities: A Delphi Study
Source: Neurocrit Care. 2023 Jul 27;40(2):645–53. doi: 10.1007/s12028-023-01799-x (PMC10959831; doi:10.1007/s12028-023-01799-x)
Supplement: Supplementary file 2 — Supplement 2: Detailed description of all 7 EPA (DOCX 42 kb) [file 12028_2023_1799_MOESM2_ESM.docx]

EPAs of Neurocritical Care: (Fit for Service)

1. **Identifying and conducting appropriate clinical (clinical-neurological) examination methods to assess neurological intensive care (NICU) patients**
2. **Performing specialized neurological diagnostic or therapeutic procedures on NICU patients**
3. **Performing general ICU-specific diagnostic and therapeutic procedures**
4. **Recognizing an emergency situation, initiate stabilization of patients and reach out for help**
5. **Transporting a NICU patient outside the NICU**
6. **Initial general management of NICU patients**
7. **Handing over neurological intensive care patients**

| **EPA 1** |  |
| --- | --- |
| **Title** | **Identifying and conducting appropriate clinical (clinical-neurological) examination methods to assess neurological intensive care (NICU) patients** |
| **Description (Specification and limitations)** | - The focus of this EPA is the use of the examination techniques the individual has acquired during medical school and residency in the setting of the NICU - This includes the implementation of suitable general physical examination and specific neurological examination techniques including the evaluation of the mental state - This also includes the collection of relevant clinical scores - The application of instrument-based diagnostics is not part of this EPA |
| **Required knowledge, skills, attitudes and experiences (KSH: Knowledge, Skills, Attitude)** | - Anatomical and neuroanatomical knowledge as well as the respective pathophysiology. - Knowledge of suitable scores for relevant (neuro)intensive care issues (FOUR, NIHSS, RASS, NPS, CAM-ICU, Hunt Hess, etc.) - Mastering clinical (clinical-neurological) examination techniques - Ability to pay attention to patient safety - Dealing professionally with patients |
| **Potential risks in case of failure** | Risk of misdiagnosis due to the use of incorrect examination methods, Incorrect execution of the examination or misinterpretation of the results  Risk of harming the patient by disregarding safety aspects  Risk of causing harm to patients |
| **Most relevant competency domains (CanMeds)** | Medical Expert  Communicator  Professional |
| **Information sources to assess progress and support summative entrustment** | Direct observation of procedural skills (DOPS)  Case-based discussions  Entries in Electronic Health Record |
| **Entrustment / supervision level expected** | Level 5 |
| **Time period to expiration if not practiced** | 5 years |

| **EPA 2** |  |
| --- | --- |
| **Title** | **Performing specialized neurologic diagnostic or therapeutic procedures on NICU patients** |
| **Description (Specification and limitations)** | - The focus of this EPA is the safe performance of diagnostic neurological procedures such as lumbar puncture (LP) (including performance while patients are in lateral lying position), taking a cerebrospinal fluid (CSF) sample from an external ventricular drain (EVD), transcranial, extracranial and orbital ultrasound examination, application of a two- or multi-channel EEG's and others. - This also includes the safe conduct of therapeutic neurological procedures such as draining the appropriate amount of CSF via EVD or lumbar drain, controlling anesthesia to achieve a burst suppression pattern on the EEG, initiating appropriate measures to reduce intracranial pressure (positioning, osmotic therapies, anesthesia, adjustment of ventilation). - The top two points also include the exclusion of contraindications and adequate communication and explanation of the corresponding measures to the patient, relatives and to the team - The subject of this EPA is not the clinical examination or the collection of scores. - Part of this EPA are not general intensive care measures, such as placing a central venous line, administration of anesthesia, etc.. |
| **Required knowledge, skills, attitudes and experiences (KSH: Knowledge, Skills, Attitude)** | - Anatomical and neuroanatomical knowledge as well as the respective pathophysiology of underlying condition - Knowledge of indications and contraindications of diagnostic and therapeutic procedures - Ability to educate patients and/or their families - Knowledge of the local Standard Operating Procedures (SOPs) for indication, implementation and documentation of the procedures - Performing instrument-based examination techniques (including instruction on the equipment) - Ensuring patient safety. - Professional handling of patients, relatives and the team.   Reference: NICU Competence catalog of the DGNI (German Neurocritical Care Society/ Deutsche Gesellschaft für Neurointensiv- und Notfallmedizin) (1) |
| **Potential risks in case of failure** | Risk of misdiagnosis due to the use of incorrect examination methods, incorrect execution of the examination or misinterpretation of the results  Risk of harming the patient by ignoring safety aspects (e.g. injuries, hemorrhages or infections during LP, ventriculitis during the collection of EVD CSF, etc.)  Risk of causing harm to patients |
| **Most relevant competency domains (CanMeds)** | Medical Expert  Communicator  Team Worker  Professional |
| **Information sources to assess progress and support summative entrustment** | Direct observation of procedural skills (DOPS)  Case-based discussions  Entries in Electronic Health Record |
| **Entrustment / supervision level expected** | Level 3 - 5 |
| **Time period to expiration if not practiced** | 4 years |

| **EPA 3** |  |
| --- | --- |
| **Title** | **Performing general ICU-specific diagnostic and therapeutic procedures** |
| **Description (Specification and limitations)** | - The focus of this EPA is the safe performance of ICU-specific diagnostic procedures and interpretation of the results. These include blood gas analysis (BGA), focused assessment with sonography in trauma (FAST) ultrasound, ultrasound examination of the extracranial vessels, performance of diagnostic tests with the ventilator, calibration of a thermodilution based Cardiac Output (CO) system - The indication and safe installation of suitable catheters (e.g. invasive arterial blood pressure measurement, central venous line, thermodilution based Cardiac Output system catheter, pleural drain, high-flow catheter, transvenous pacemakers). - Indication and safe application of organ-supporting therapies (e.g. intubation and adjustment of the ventilator, continuous renal replacement therapy, placement of a temporary pacemaker) - The above points also include the exclusion of contraindications, obtaining informed consent from patients or legal representatives and adequate communication with patients, relatives and the team |
| **Required knowledge, skills, attitudes and experiences (KSH: Knowledge, Skills, Attitude)** | - Anatomical and neuroanatomical knowledge as well as the respective pathophysiology - Knowledge of indications and contraindications of diagnostic and therapeutic procedures. - Knowledge of the local Standard Operating Procedures (SOPs) for indication, implementation and documentation of the procedures - Mastering instrument-based examination techniques (including instruction on the equipment) - Ability to pay attention to patient safety. - Professional handling of patients, relatives and the team   Reference: NICU Competence catalog of the DGNI (German Neurocritical Care Society / Deutsche Gesellschaft für Neurointensiv- und Notfallmedizin) (1) |
| **Potential risks in case of failure** | Risk of misdiagnosis due to the use of incorrect examination methods, incorrect execution of the examination or misinterpretation of the results  Risk of harming the patient by ignoring safety aspects (e.g. incorrect intubation, iatrogenic pneumothorax, hemorrhagic shock or major bleeding, cardiac arrhythmia).  Risk of causing harm to patients. |
| **Most relevant competency domains (CanMeds)** | Medical Expert  Communicator  Team Worker  Professional |
| **Information sources to assess progress and support summative entrustment** | Direct observation of procedural skills (DOPS)  Case-based discussions  Entries in Electronic Health Record |
| **Entrustment / supervision level expected** | Level 3 - 5 |
| **Time period to expiration if not practiced** | 2 years |

| **EPA 4** |  |
| --- | --- |
| **Title** | **Recognizing an emergency situation, initiate stabilization of patients and reach out for help** |
| **Description (Specification and limitations)** | - The focus of this EPA is the recognition of an emergency situation in the intensive care setting (this includes neurological emergencies such as increase in intracranial pressure, new neurological deficits, loss of consciousness, etc., as well as intensive care medical emergencies such as acute circulatory insufficiency, respiratory insufficiency, etc.) - This EPA includes taking the first diagnostic steps to identify the cause - Initiation of the first steps to stabilize the patient - Communication with the team and call for help |
| **Required knowledge, skills, attitudes and experiences (KSH: Knowledge, Skills, Attitude)** | - Knowledge of physiology, pathophysiology and anatomy of the nervous system and other organs - Knowledge of suitable algorithms for emergency situations - Mastering physical (clinical-neurological) examination techniques to establish differential diagnoses - Mastering diagnostic and therapeutic procedures of critical care medicine - Leading the team during an emergency situation - Communication with the patient and the team |
| **Potential risks in case of failure** | Risk of misdiagnosis due to the use of incorrect examination methods, incorrect execution of the examination or misinterpretation of the results  Risk of harming the patient due to inappropriate therapeutic measures or miscommunication  Risk of causing harm to patients |
| **Most relevant competency domains (CanMeds)** | Medical Expert  Communicator  Team Worker  Leader  Professional |
| **Information sources to assess progress and support summative entrustment** | Direct observation of procedural skills (DOPS)  Multisource feedback (MSF) or 360° Evaluation  Case-based discussions  Entrustment-based discussions |
| **Entrustment / supervision level expected** | Level 3 and 4. |
| **Time period to expiration if not practiced** | 2 years |

| **EPA 5** |  |
| --- | --- |
| **Title** | **Transporting an NICU patient outside of the NICU** |
| **Description (Specification and limitations)** | - Preparing an intrahospital transport of an ICU patient (premedication if indicated, appropriate monitoring, preparation of useful medication or equipment for transport) - Carrying out the transport (including handling of equipment, e.g. manual ventilation if indicated). - Dealing with emergencies during transport. - Hand-over of the patient to other medical professionals at - Communicating with the team and obtaining help are part of this EPA. |
| **Required knowledge, skills, attitudes and experiences (KSH: Knowledge, Skills, Attitude)** | - Knowledge of suitable algorithms for emergency situations - Knowledge of the pharmacological properties of vasoactive substances, sedatives and other emergency drugs frequently used - Mastering use of equipment during transport. - Leading the team in an emergency situation - Communication with patients and the team - Ability to hand over the most important medical information in a structured manner |
| **Potential risks in case of failure** | Risk of missing emergency equipment necessary to manage complications during transport  Risk of harming the patient due to failure to use appropriate therapeutic measures or miscommunication (e.g. during handover).  Risk of causing harm to patients |
| **Most relevant competency domains (CanMeds)** | Medical Expert  Communicator  Team Worker  Leader  Professional |
| **Information sources to assess progress and support summative entrustment** | Direct observation of procedural skills (DOPS)  Case-based discussions  Entrustment-based discussions |
| **Entrustment / supervision level expected** | Level 4 and 5. |
| **Time period to expiration if not practiced** | 2 years |

| **EPA 6** |  |
| --- | --- |
| **Title** | **Initial general management of NICU patients** |
| **Description (Specification and limitations)** | - The focus of this EPA is to perform diagnostic tests to specify the admission diagnosis (this also includes medical history taking and third-party medical history taking, ordering additional diagnostics such as imaging, laboratory tests, electrophysiology, etc.). - Implementation of the first therapeutic steps (both symptom control and treatment of the etiology) for the most common neurological disorders requiring intensive care treatment. - The specific clinical-neurological examination of the intensive care patient is not the subject of this EPA. - Communicating with the team and leading it - Formulation of clinical questions for specific examination methods |
| **Required knowledge, skills, attitudes and experiences (KSH: Knowledge, Skills, Attitude)** | - Knowledge of the clinical presentation, pathophysiology, epidemiology, necessary diagnostics and initial therapy of the most common (neuro)intensive medical diseases - Knowledge of indications, sensitivity/specificity and diagnostic value for specific additional examinations - Clinical reasoning and development of a diagnostic and therapeutic plan - Communication with patients, caregivers and the team; the latter in an interprofessional and interdisciplinary fashion. |
| **Potential risks in case of failure** | Risk of delaying the correct diagnosis, possibly resulting in harm of the patient  Risk of miscommunication within the team and with neighboring disciplines |
| **Most relevant competency domains (CanMeds)** | Medical Expert  Communicator  Professional |
| **Information sources to assess progress and support summative entrustment** | Multisource feedback (MSF) or 360° Evaluation  Chart-Stimulated Recall  Case-based discussions  Entries in Electronic Health Record |
| **Entrustment / supervision level expected** | Level 4 and 5. |
| **Time period to expiration if not practiced** | 3 years |

| **EPA 7** |  |
| --- | --- |
| **Title** | **Handing over neurological intensive care patients**. |
| **Description (Specification and limitations)** | - The focus of this EPA is to convey important information to the treatment team at handover - This includes both the verbal handover and ongoing documentation in the (electronic) chart |
| **Required knowledge, skills, attitudes and experiences (KSH: Knowledge, Skills, Attitude)** | - Ability to think critically - Ability to provide structured information - Knowledge of structured handover tools - Addressing information gaps or misperceptions in the team |
| **Potential risks in case of failure** | Risk of harm due to repetition of examinations or lack of information within the treatment team.  Risk of negative team dynamics |
| **Most relevant competency domains (CanMeds)** | Medical Expert  Communicator  Professional |
| **Information sources to assess progress and support summative entrustment** | Direct observation of procedural skills (DOPS)  Multisource feedback (MSF) or 360° Evaluation  Chart-Stimulated Recall  Case-based discussions  Entries in Electronic Health Record |
| **Entrustment / supervision level expected** | Level 4 and 5 |
| **Time period to expiration if not practiced** | 5 years |

Level 1: the learner is allowed to be present and observe, not to enact an EPA

Level 2: the learner is allowed to execute the EPA with direct, pro-active supervision, present in the room

Level 3: the learner is allowed to carry out the EPA without a supervisor in the room, but quickly available if needed, i.e. with indirect, reactive, supervision

Level 4: the learner is allowed to work unsupervised

Level 5: the learner is allowed to provide supervision to more junior learners.

1. Niesen WD, Gahn G, Salih F, Biesalski AS, Czorlich P, Dohmen C, et al. Curriculum zur Zusatzweiterbildung bzw. Erlangung der Zusatzbezeichnung Intensivmedizin für Fachärzt*innen der Neurologie und Neurochirurgie. DGNeurologie. 2023;6(1):5-12.
