# Supplementary material for: Fit for Service: Preparing Residents for Neurointensive Care with Entrustable Professional Activities: A Delphi Study
Source: Neurocrit Care. 2023 Jul 27;40(2):645–53. doi: 10.1007/s12028-023-01799-x (PMC10959831; doi:10.1007/s12028-023-01799-x)
Supplement: Supplementary file 3 — Supplement 3: Table with detailed scoring for the EQual instrument per Item and EPA (DOCX 17 kb) [file 12028_2023_1799_MOESM3_ESM.docx]

| **Items** | **EPA 1** | **EPA 2** | **EPA 3** | **EPA 4** | **EPA 5** | **EPA 6** | **EPA 7** |
| --- | --- | --- | --- | --- | --- | --- | --- |
| This EPA has a clearly defined beginning and end | 5 | 5 | 5 | 5 | 5 | 4 | 5 |
| This EPA is independently executable to achieve a defined clinical outcome | 4 | 4 | 5 | 3,5 | 4 | 3,5 | 4 |
| This EPA is specific and focused | 5 | 5 | 5 | 5 | 5 | 4 | 5 |
| This EPA is observable in process | 4 | 5 | 5 | 5 | 5 | 3,5 | 5 |
| This EPA is measurable in outcome | 5 | 5 | 5 | 5 | 5 | 4 | 5 |
| This EPA is clearly distinguished from other EPAs in the framework | 5 | 5 | 5 | 5 | 4 | 5 | 5 |
| This EPA describes work that is essential and important to the profession | 5 | 5 | 5 | 5 | 5 | 5 | 5 |
| Performing this EPA leads to recognized output or outcome of labor | 5 | 5 | 5 | 5 | 5 | 5 | 5 |
| The performance of this EPA in clinical practice is restricted to qualified personnel | 4 | 5 | 5 | 5 | 5 | 5 | 5 |
| This EPA addresses professional work that is suitable for entrustment | 5 | 5 | 5 | 5 | 5 | 5 | 5 |
| This EPA requires the application of knowledge, skills, and/or attitudes (KSAs) acquired through training | 4 | 5 | 5 | 5 | 5 | 5 | 4 |
| This EPA involves application and integration of multiple domains of competence | 5 | 5 | 5 | 5 | 5 | 5 | 5 |
| The EPA title describes a task, not qualities or competencies of a learner | 4 | 5 | 5 | 5 | 5 | 4 | 5 |
| This EPA describes a task and avoids adjectives (or adverbs) that refer to proficiency | 5 | 5 | 5 | 5 | 5 | 5 | 5 |
| Total Mean (Standard Deviation) | 4,64 (± 0,50) | 4,93 (± 0,27) | 5,0 (± 0,0) | 4,89 (± 0,40) | 4,86 (± 0,36) | 4,5 (± 0,62) | 4,86 (± 0,36) |

Mean EQual Scores (of two raters) for the 14 items. Total EQual scores with Standard Deviations

EPA 1: Identifying and conducting appropriate clinical (clinical-neurological) examination methods to assess neurological intensive care (NICU) patients

EPA 2: Performing specialized neurological diagnostic or therapeutic procedures on NICU patients

EPA 3: Performing general ICU-specific diagnostic and therapeutic procedures

EPA 4: Recognizing an emergency situation, initiate stabilization of patients and reach out for help

EPA 5: Transporting a NICU patient outside the NICU

EPA 6: Initial general management of NICU patients

EPA 7: Handing over neurological intensive care patients
