# Supplementary material for: Fit for Service: Preparing Residents for Neurointensive Care with Entrustable Professional Activities: A Delphi Study
Source: Neurocrit Care. 2023 Jul 27;40(2):645–53. doi: 10.1007/s12028-023-01799-x (PMC10959831; doi:10.1007/s12028-023-01799-x)
Supplement: Supplementary file 4 — Supplement 4: Table with values for the adjusted interrater reliability after removing items 2, 6 and 14 (DOCX 14 kb) [file 12028_2023_1799_MOESM4_ESM.docx]

| **EPA Titles** | **Krippendorff's 𝛼** |
| --- | --- |
| 1. Identifying and conducting appropriate clinical (clinical-neurological) examination methods to assess neurological intensive care (NICU) patients | 0,70 |
| 1. Performing specialized neurological diagnostic or therapeutic procedures on NICU patients | 1,0 |
| 1. Performing general ICU-specific diagnostic and therapeutic procedures | 1,0 |
| 1. Recognizing an emergency situation, initiate stabilization of patients and reach out for help | 1,0 |
| 1. Transporting a NICU patient outside the NICU | 1,0 |
| 1. Initial general management of NICU patients | 0,73 |
| 1. Handing over neurological intensive care patients | 1,0 |
| **Total** | 0,80 |

Interrater Reliability without Items 2, 6 and 14
